# Novel interaction between Alzheimer’s disease-related protein presenilin 1 and glutamate transporter 1

**DOI:** 10.1038/s41598-018-26888-2

**Published:** 2018-06-07

**Authors:** Katarzyna Marta Zoltowska, Masato Maesako, Joshua Meier, Oksana Berezovska

**Affiliations:** 1 MassGeneral Institute for Neurodegenerative Disease, Massachusetts General Hospital, Harvard Medical School, Department of Neurology, 114 16th Street, 02129 Charlestown, MA USA; 2 Laboratory of Preclinical Testing of Higher Standard, Neurobiology Center, Nencki Institute of Experimental Biology, 3 Pasteur Street, 02-093 Warsaw, Poland

## Abstract

Neuronal hyperactivity is one of the earliest events observed in Alzheimer’s disease (AD). Moreover, alterations in the expression of glutamate transporters have been reported to exacerbate amyloid pathology and cognitive deficits in transgenic AD mouse models. However, the molecular links between these pathophysiological changes remain largely unknown. Here, we report novel interaction between presenilin 1 (PS1), the catalytic component of the amyloid precursor protein-processing enzyme, γ-secretase, and a major glutamate transporter-1 (GLT-1). Our data demonstrate that the interaction occurs between PS1 and GLT-1 expressed at their endogenous levels *in vivo* and *in vitro*, takes place in both neurons and astrocytes, and is independent of the PS1 autoproteolysis and γ-secretase activity. This intriguing discovery may shed light on the molecular crosstalk between the proteins linked to the maintenance of glutamate homeostasis and Aβ pathology.

## Introduction

Alzheimer’s disease (AD) is a neurodegenerative disorder, characterized neuropathologically by progressive formation of intracellular neurofibrillary tangles (NFTs), extracellular amyloid β (Aβ) plaques and synaptic dysfunctions. Several lines of evidence indicate the existence of a link between the observed pathology and neuronal hyperactivity stemming from the imbalance in the inhibitory and excitatory inputs at the synapses in the brain^1,2^. Glutamate is the major excitatory neurotransmitter in the CNS^3,4^. The level of glutamate at the synapses is controlled by the concomitant action of neurons and astrocytes. The latter play a central role in the cellular phase of AD^5^.

A number of studies have reported that people with genetic risk for AD, cognitively normal individuals with evidence of Aβ accumulation, and those with early stages of AD exhibit hyperactivation and impaired deactivation of the default-mode network during hippocampal memory-encoding^6–10^. In addition, it has been shown that network hyperactivity and alterations in glutamate transporters expression precede amyloid plaque formation in AD mouse models^11,12^, and that glutamate dysregulation occurs prior to and positively correlates with the cognitive decline in human subjects^13,14^. Furthermore, clinical observations suggest that familial AD (fAD) patients, including those with presenilin 1 (PS1) mutations, may have higher incidence of epileptic seizures^15^ and that individuals diagnosed with epilepsy in childhood present more severe amyloid plaque deposition as middle-aged adults^16^. These observations hint towards the potential cross-talk between PS1/amyloid deposition and hyperactivity/Glu dyshomeostasis in the brain. Intriguingly, in an unbiased mass spectrometry search for novel PS1 binding partners, we discovered that glutamate transporter 1 (GLT-1, alternative names: EAAT2, SLC1A2), the major glutamate transporter in the central nervous system, interacts with the PS1 large cytosolic loop located between the 6^th^ and 7^th^ transmembrane domains.

Since 80–90% of the GLT-1 transporter is expressed in astrocytes^17^, it was first described as an astrocytic glutamate transporter, signifying the importance of astrocytes in glutamate uptake and neuroprotection^18,19^. However, GLT-1, more specifically GLT1a, expression and activity have now also been demonstrated in neurons, where it plays an important role in axonal glutamate uptake^17,20–23^. The functionally active GLT-1 exists as a dynamic homo-multimer that freely diffuses at the plasma membrane where it undergoes an allosteric conformational change upon glutamate and Na^+^ binding. This conformational shift enables the transport of glutamate from the extracellular space into the cytoplasm^24–29^. GLT-1 knock-out mice demonstrate severe impairments, including increased hyperactivity and shorter lifespan^21,22^. On the other hand, acute activation of GLT-1 can reshape the brain’s metabolism and synaptic signaling, as shown by 2-deoxy-2-[^18^F]fluoro-D-glucose ([^18^F]FDG) positron emission tomography^30^. Given the importance of GLT-1, its aberrant expression and/or impaired function in the brain might have detrimental effects, including those leading to excitotoxicity and neurodegeneration. Despite the comprehensive data demonstrating the importance of GLT-1 in the brain, little is known about factors modulating its cell surface expression and function, and how these might be altered in AD. The current study reveals a molecular link between PS1 and GLT-1, presenting a rationale for further evaluation of the PS1-GLT-1 binding as potential novel therapeutic target.

## Results

### Mass spectrometry screen reveals GLT-1 as a novel PS1 binding partner

To search for novel presenilin 1 (PS1)-interacting proteins, we performed an unbiased mass spectrometry (MS) screen of mouse brain lysates, prepared using 1% Triton X-100 (TX-100) detergent. Glutathione S-transferase (GST)-tagged peptides, corresponding to the PS1 N-terminus (NT; amino acids (aa) 1-80), PS1 loop between the 1^st^ and 2^nd^ transmembrane helices (L1-2; amino acids, aa 98–134), and PS1 loop located between the 6^th^ and 7^th^ transmembrane helices (L6-7; aa 263–376) were used as baits. GST alone pull-down served as a negative control. Glutamate transporter 1 (GLT-1) was identified as one of the novel PS1-binding partners. The PS1-GLT-1 binding was revealed when the PS1 L6-7, but not the PS1 NT or L1-2 peptides were used for the pull-down, and was detected in the same excised gel slice of 60kD-70kD bands that also contained synaptotagmin 1 (Syt1), reported in^31^.

### PS1 interacts with GLT-1 *in vivo* and in astrocytes and neurons *in vitro*

**Figure 1:**
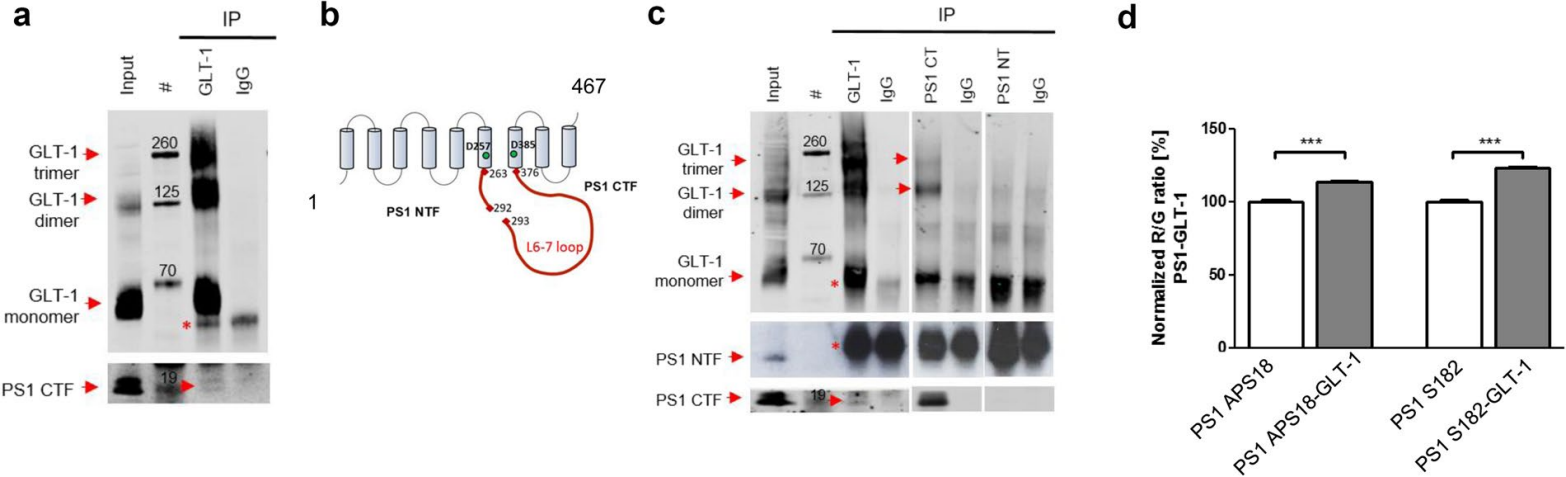
PS1-GLT-1 complexes are present in mouse brain. (**a**,**c**) The representative western blots demonstrate co-immunoprecipitation of the PS1-GLT-1 complexes from mouse brain lysates prepared using (**a**) 1% CHAPSO or (**c**) 1% TX-100 buffer. The arrows indicate bands corresponding to the proteins co-immunoprecipitated using the respective antibodies. IgG heavy and light chain bands are marked by red asterisks. N = 3 independent experiments for each condition. (**b**) The schematic representation of the PS1 NTF/CTF assembly. The large cytoplasmic loop containing the autoproteolysis site is marked in red. Aspartate residues responsible for the γ-secretase activity are marked with the green circles. (**d**) Spectral FRET detection of the PS1-GLT-1 interaction in mouse brain sections. Two different antibodies, anti-PS1 loop (APS18) and anti-PS1 CT (S182), were used for the PS1 detection; AB1783 (Millipore) antibody was used to label GLT-1. An increase in the R/G ratio signals presence of FRET, indicative of the interaction between the two proteins. Two-tailed, unpaired Student’s t-test, ***p < 0.001; N = 3 independent experiments.

To test whether the PS1-GLT-1 interaction may occur *in vivo* between the proteins expressed at their endogenous levels, the PS1-GLT-1 complexes were immunoprecipitated using anti-GLT-1 antibody (Abcam, ab41621) from mouse brains lysed in 1% CHAPSO buffer. Western blot analysis using the anti-PS1 loop antibody for detection demonstrated the presence of PS1 C-terminal fragments (PS1-CTF) in the immunoprecipitated fraction (Fig. 1a), suggestive of the binding between the GLT-1 and PS1 proteins at their endogenous levels of expression in the mouse brain. Next, to detail whether GLT-1 interacts with the N- and/or C-terminal PS1 fragment (NTF or CTF, respectively, Fig. 1b), we conducted PS1-GLT-1 co-immunoprecipitation from mouse brain lysates prepared using 1% TX-100 as a detergent, known to disrupt the PS1-NTF/CTF assembly^32^. Anti-PS1-NT or anti-PS1-CT antibodies were used for the pull-down. GLT-1 was selectively co-immunoprecipitated with PS1 when the anti-PS1-CT, but not the anti-PS1-NT, antibody was used (Fig. 1c). Considering that GLT-1 was pulled down using the PS1 L6-7 fragment (aa 263–376) as a bait in the initial MS screen, and that PS1 undergoes auto-proteolysis at aa 292/293 during maturation (Fig. 1b), the PS1-GLT-1 interaction site can be narrowed to the amino acids 293–376 within the PS1 sequence.

To confirm the co-immunoprecipitation data using a different approach, and to demonstrate that the PS1-GLT-1 interaction occurs in intact cells when the proteins physiological environment is not disrupted, we employed spectral Förster resonance energy transfer (FRET) assays in mouse brain tissue. The tissue sections were immunostained with anti-PS1 and anti-GLT-1 (Millipore, AB1783) antibodies, followed by AF488 and Cy3-labelled secondary antibodies, respectively. Anti-PS1 antibodies from two different vendors, directed against PS1 loop domain (APS18) or PS1 C-terminus (S182), were used in the experiments. Anti-GLT-1 antibody was omitted in the samples, which served as negative FRET controls. Increased R/G ratio, suggestive of the energy transfer and hence <5 nm distance between the fluorophores consistent with the proteins’ interaction, was observed in the PS1-GLT-1 double-immunostained mouse brain tissue (Fig. 1d). Together, these data provide strong evidence of the endogenous PS1-GLT-1 interaction in the mouse brain.

**Figure 2:**
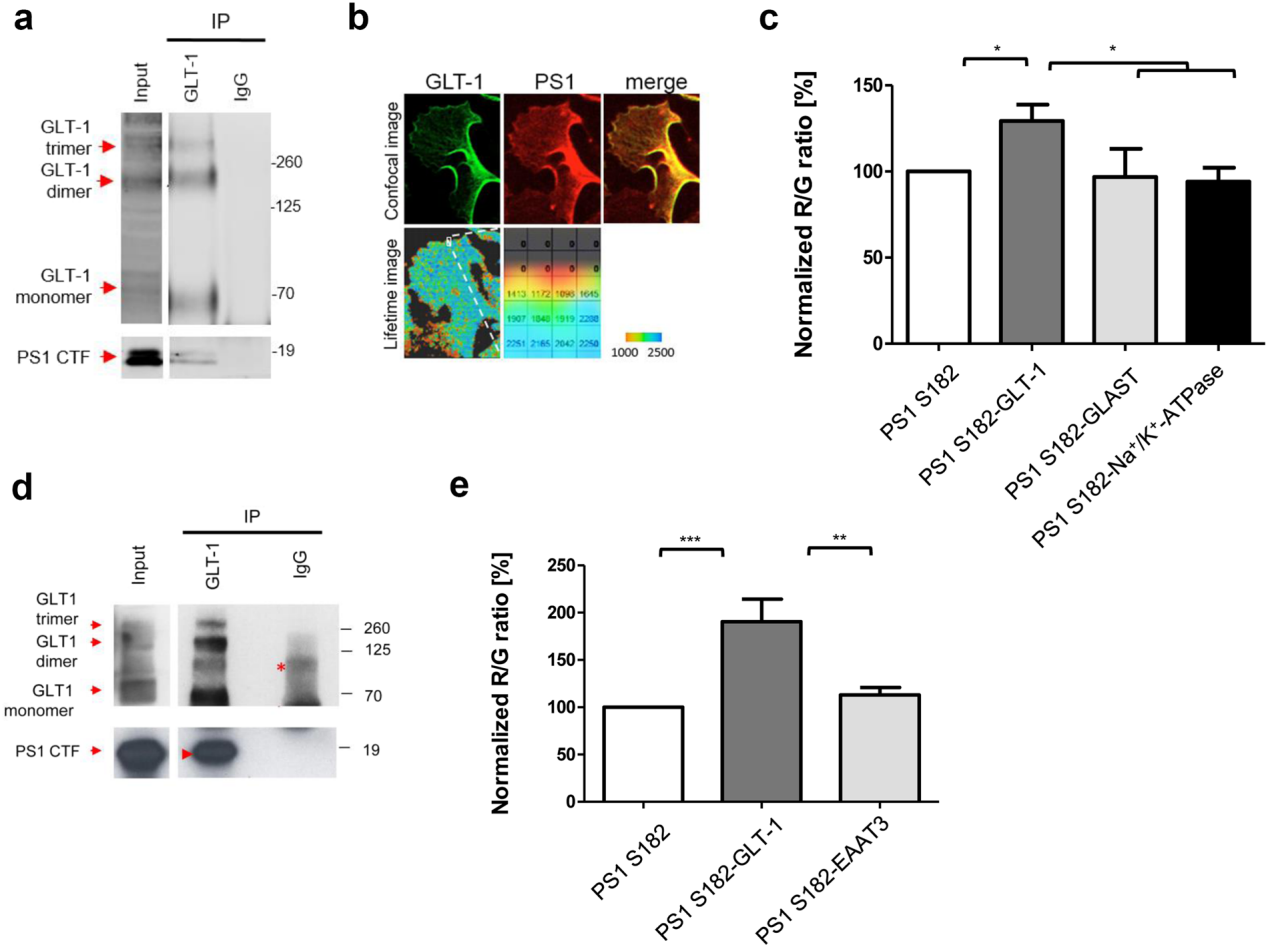
PS1 interacts with GLT-1 transporter in primary astrocytes and neurons. (**a**,**d**) The representative western blots demonstrate coimmunoprecipitation of the PS1-GLT-1 complexes from mouse primary astrocyte (**a**) or neuron (**d**) lysates prepared using 1% CHAPSO buffer. The ab41621 (Abcam) anti-GLT-1 antibody was used for pull-down. The arrows indicate bands corresponding to the proteins co-immunoprecipitated using the respective antibodies. Red asterisks mark IgG heavy and light chains and nonspecific bands. N = 3 independent experiments for each condition. (**b**) Fluorescence lifetime imaging assay (FLIM) of the PS1-GLT-1 proximity in primary astrocytes. Top panel shows astrocyte immunostained with anti-GLT-1 and anti-PS1 antibodies. Bottom panels show color-coded FLIM images of the donor fluorophore lifetimes in the cell. Orange-red pixels, corresponding to the shorter fluorescence lifetimes, are indicative of the closest proximity between the fluorophore-labeled PS1 and GLT-1, revealing PS1-GLT-1 binding sites. The colorimetric scale shows AF488 fluorescence lifetime in picoseconds. (**c**,**e**) Spectral FRET detection of the PS1 interaction with glutamate transporters in mouse primary astrocytes (**c**) and neurons (**e**). Anti-PS1 CT (S182), anti-GLT-1 (AB1783), anti-GLAST (MABN794), anti-EAAT3 (MAB1587) and anti-Na/K ATPase antibodies were used for the analysis. The presence of the interaction between two proteins is demonstrated by the increased R/G ratio in the double-immunostained cells, compared to the samples where one primary antibody is omitted (FRET negative controls). The presence of FRET signal was only observed in PS1-GLT-1 double-immunostained cells. One-way Anova with Bonferroni post hoc test, ***p < 0.001; N = 6 and N = 3 independent experiments for astrocytes and neurons, respectively.

Although the majority of GLT-1 in the brain is expressed in astrocytes^17,25^, its neuronal expression has also been reported^17,20^. Therefore, to specify whether PS1 interacts with astrocytic or neuronal GLT-1, we performed a series of co-immunoprecipitation, spectral FRET and fluorescence lifetime imaging microscopy (FLIM) experiments assessing the formation of the PS1-GLT-1 complexes in *in vitro* cultured primary astrocytes and neurons (Fig. 2). Successful immunoprecipitation of the PS1 CTF after pull-down with the anti-GLT-1 (Abcam, ab41621) antibody was observed in both astrocytes (Fig. 2a) and in neurons (Fig. 2d).

These findings were further verified using complementary approaches – FLIM and spectral FRET assays in intact cells (Fig. 2b,c,e). Primary astrocytes immunostained with AF488-labeled GLT-1 (Millipore, AB1783) and Cy3-labeled PS1 antibodies show co-localization of the two proteins (Fig. 2b top panels). High-resolution FLIM^31,33^ generated a color-coded image of the double-immunostained astrocyte (Fig. 2b bottom panels), revealing that PS1 comes into the closest proximity with GLT-1 at/near the plasma membrane (red pixels, shortest lifetimes signaling FRET). Spectral FRET analysis of both primary astrocytes and neurons verified the FLIM data and showed an increased R/G ratio (presence of FRET) in the PS1-GLT-1 double-immunostained cells compared to the single-immunostained negative FRET controls (Fig. 2c,e). Of note, no FRET was detected between the immunolabelled PS1 and Na^+^/K^+^ ATPase, another abundant cell surface protein, confirming the specificity of the detected PS1-GLT-1 interaction.

GLT-1 shares some degree of the sequence homology with other glutamate transporters. Thus, we next evaluated whether PS1 might bind to other glutamate transporters, such as GLAST or EAAT3, in either astrocytic or neuronal cells, respectively. To this end, we performed FRET experiments in astrocytes immunostained with antibodies recognizing PS1 and GLAST, and in neurons immunostained with antibodies targeting PS1 and EAAT3. No significant increase in the R/G ratio was recorded in these cells (Fig. 2c,e), suggesting that PS1 selectively and specifically interacts with the GLT-1.

### PS1-GLT-1 interaction is independent of PS1 autoproteolysis and γ-secretase activity

To characterize the molecular determinants of the PS1-GLT-1 binding, we investigated whether PS1 autoproteolysis or activity are needed for the interaction. The immature PS1 holoprotein travels through the secretory pathway, where it undergoes autoproteolysis to yield mature PS1 NTF/CTF heterodimer (Fig. 1b)^34^. The autoproteolysis step is a critical event for PS1 to acquire its proteolytic activity, with only the PS1 ∆e9 mutant being an exception to this rule^34,35^. The autoproteolytic cleavage relies on the two aspartates in the positions 257 and 385, and the introduction of a single amino acid substitution in any of those residues (D257A or D385A) abolishes the autoproteolysis, and therefore abolishes PS1/γ-secretase activity^36^.

**Figure 3:**
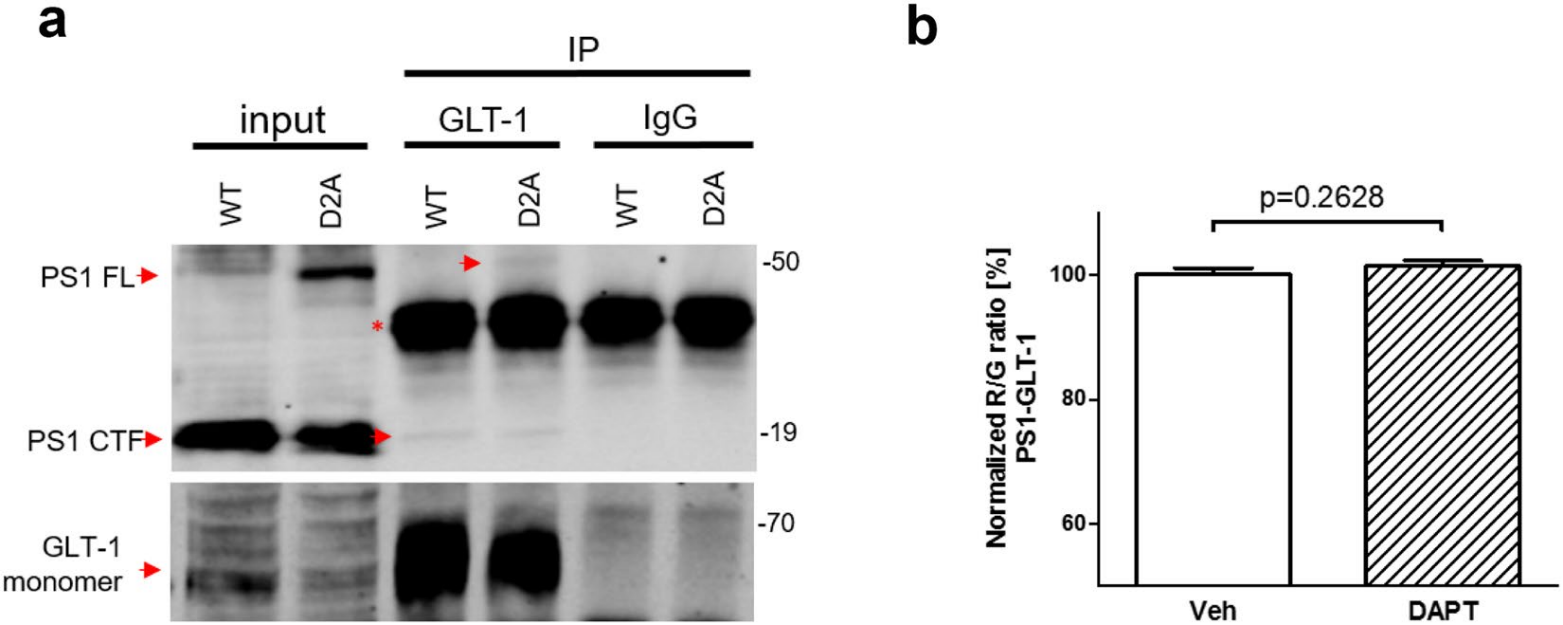
PS1/γ-secretase activity is not needed for the PS1-GLT-1 interaction (**a**) PS1-GLT-1 complexes were immunoprecipitated from Chinese hamster ovary (CHO) cells stably expressing PS1 wild type (WT) or PS1.D257A mutant (D2A), and transiently transfected with GLT-1-encoding plasmid. The cells were lysed in 1% CHAPSO. The red arrows indicate that both PS1 CTF and PS1 FL were successfully co-immunoprecipitated using AB1783 anti-GLT-1 antibody. IgG heavy chain bands are marked by red asterisk. N = 3 independent experiments. (**b**) Spectral FRET analysis of the endogenous PS1-GLT-1 interaction in primary astrocytes treated for 16 hours with vehicle or 1 μM γ-secretase inhibitor DAPT. Two-tailed, unpaired Student’s t-test, n = 165–232; n = number of astrocytes analyzed in three independent experiments.

To determine whether the PS1/γ-secretase activity is required for the interaction, we immunoprecipitated PS1-GLT-1 complexes from Chinese hamster ovary (CHO) cell lines stably expressing PS1.wt or PS1.D257A, and transiently transfected with GLT-1 (Fig. 3a). Successful pull-down of the PS1-CTF as well as PS1 full length (FL) from both cell lines using anti-GLT-1 antibody (Abcam, ab41621) indicated that the PS1 autoproteolysis and γ-secretase activity are not needed for the binding to GLT-1.

Next, to corroborate these findings in intact cells using a complementary approach, we analyzed PS1-GLT-1 interaction by spectral FRET in vehicle vs. γ-secretase inhibitor N-[(3,5-Difluorophenyl)acetyl]-L-alanyl-2-phenyl]glycine-1,1-dimethylethyl ester (DAPT)-treated primary astrocytes (Fig. 3b). Consistent with the co-IP experiments from CHO D257A cell line, DAPT did not prevent PS1-GLT-1 binding. These data provide further evidence that the PS1-GLT-1 interaction is independent of the PS1/γ-secretase activity.

## Discussion

Presenilin 1 (PS1) is the catalytic component of γ-secretase, responsible for the final cut of amyloid precursor protein (APP) that releases amyloid β (Aβ) peptides of different lengths. It has been recently reported that PS1 may be involved in the modulation of neurotransmitter release at the synapse via direct interaction with ryanodine receptors (RyR)^37^ and synaptic vesicle release machinery protein, synaptotagmin 1, in neurons^31,33^. Here we report the discovery of the PS1 interaction with the glutamate transporter GLT-1 in both neurons and astrocytes, which may suggest a novel role of PS1 in regulation of the glutamate uptake. Interestingly, we found that PS1 D257A mutation and γ-secretase inhibitor DAPT do not prevent PS1 interaction with GLT-1. This raises an intriguing possibility that PS1, in addition to previously reported γ-secretase activity-independent role in regulation of the calcium homeostasis, protein trafficking and apoptosis^38–41^, may have yet another activity-independent role in the modulation of glutamate balance and homeostasis at the synapse.

Interestingly, PS1 does not seem to interact with either neuronal EAAT3 or astrocytic GLAST transporters. The direct PS1-GLT-1 binding might provide a missing link between the PS1-mediated Aβ pathology^42,43^ and impaired glutamate transport reported during the early stages of the Alzheimer’s disease^12–14^. The level of glutamate uptake, measured by D- and L-[^3^H] aspartate binding assay in membrane preparations from the brain midfrontal cortex, as well as the GLT-1 mRNA and protein expression levels are markedly reduced in AD brains^44–46^ and our unpublished data). In addition, biochemical assessment of the sporadic AD and control brain tissue showed aberrant accumulation of detergent-insoluble GLT-1 in AD^47^. These findings are indicative of the aberrations of either neuronal or astrocytic, or both, glutamate uptake systems in AD. However, detailed functional studies are needed to determine the causality of altered GLT-1 expression/function and Aβ/tau/AD pathology. The detrimental impact of Aβ, and Aβ42 in particular, on GLT-1 subcellular localization and ability to clear glutamate has been reported^48–50^. On the other hand, it is also possible that the reduced GLT-1 expression, in addition to the diminished glutamate uptake^13,14^ may affect the PS1-dependent amyloid precursor protein (APP) processing due to diminished PS1-GLT-1 interaction, thus contributing to AD pathology. In line with this hypothesis, genetic reduction of GLT-1 expression in AD mouse models at an early age has been demonstrated to accelerate the amyloid pathology and worsen cognitive decline^51^. On the other hand, pharmacological upregulation of GLT-1 levels has been shown to provide neuroprotection^52,53^. Since pathogenic conformational changes in PS1 that are linked to elevated Aβ42/40 ratio precede amyloid deposition in the brain^54,55^, it is possible that GLT-1 may be yet another positive regulator of the PS1 conformation (unpublished observation), influencing the PS1 subdomain arrangement, similarly to that of neuronal synaptotagmin 1 (Syt1) and N-cadherin^31,33,56,57^. Although the predominant view of the AD pathological cascade remains primarily ‘neurocentric’, contribution of astrocytes, the most abundant fraction of glial cell types in the adult brain^58^, cannot be underappreciated and warrants greater attention.

In summary, the discovery of the interaction between the Alzheimer’s disease-related protein PS1 and the major glutamate transporter (GLT-1) may provide a missing link between the amyloid pathology and aberrant glutamate transport in AD. It may open new avenues for the research focusing on the impairments in glutamate homeostasis and on the contribution of astrocytes to the total Aβ pool in the disease.

## Methods

### Mass spectrometry screen

The glutathione S-transferase (GST)-tagged PS1-corresponding peptides were prepared by subcloning DNA fragments encoding human presenilin 1 N-terminus (PS1 NT) (amino acids, aa, 1–80), PS1 loop 1–2 (aa 98–134), and PS1 loop 6–7 (aa 263–376) into the pGEX-6P-2 plasmid (GE Healthcare Life Sciences, Pittsburgh, PA). *Escherichia coli* BL21 (*fhuA2 [lon] ompT gal [dcm] ΔhsdS*) cells were transformed with the respective PS1 fragments-encoding plasmids. 1 mM isopropyl-D-thiogalactoside (IPTG) was used to induce protein expression. To extract the overexpressed protein, the bacterial cultures were centrifuged at 3,500xg for 20 minutes at 4 °C, resuspended in TBST and sonicated. 15-minute centrifugation at 14,000xg at 4 °C was performed to clarify the lysates, which were then applied to the glutathione-sepharose 4B beads. Then the beads coupled with the respective peptides were incubated with mouse brain lysates prepared in 1% Triton X-100-based buffer (50 mM HEPES, 100 mM NaCl, pH 7.4 and 1% TX-100) supplemented with HALT protease and phosphatase inhibitor cocktails (ThermoScientific, Waltham, MA). The beads coupled with the recombinant GST peptide were used as a control. The elution was performed with 2xLDS NuPage sample buffer (ThermoScientific, Waltham, MA). The eluted fractions were separated by electrophoresis on 4–12% 1.5 mm Bis-Tris NuPage gels (ThermoScientific, Waltham, MA), which were subsequently stained with Coomassie blue (ThermoScientific, Waltham, MA), following the manufacturer’s protocol. Several bands abundant in the PS1 fragments-coupled fractions were excised, and analyzed at Harvard University’s Taplin Biological Mass Spectrometry Facility (https://taplin.med.harvard.edu/).

### Cell culture

Cortical primary astrocytic cultures were prepared from mouse pups at P1-3, as described previously^59^.The cells were maintained in high-glucose DMEM supplemented with 10% FBS and 1% penicilin/streptomycin mix (ThermoScientific, Waltham, MA) in a 37 °C, 5% CO2 incubator.

Mixed cortical and hippocampal primary neuronal cultures were prepared using established protocols^31,33^. Briefly, the brains were dissected from mouse embryos at the 14–16 gestation day (Charles River Laboratories, Wilmington, MA). The neurons were dissociated using Papain Dissociation System (Worthington Biochemical Corporation, Lakewood, NJ, USA). The neuronal cultures were maintained for 13–15 days *in vitro* (DIV) in Neurobasal medium containing 2% B27 supplement, 1% GlutaMax and 1% penicilin/streptomycin mix (ThermoScientific, Waltham, MA) in 37 °C, 5% CO_2_ incubator. All experimental procedures using mice were carried out in accordance with the NIH guidelines for the use of animals in experiments and were approved by the Massachusetts General Hospital Animal Care and Use Committee.

Chinese hamster ovary (CHO) cell lines stably expressing APP and wild type PS1 or PS1.D257A mutant (D2A) were kind gifts from Dr. Dennis Selkoe (BWH, Boston). The cells were maintained in OPTIMEM medium supplemented with 5% FBS, and 200 μg/ml G418 and 250 μg/ml zeocin, as selection antibiotics (ThermoScientific, Waltham, MA) in a 37 °C, 5% CO_2_ incubator.

### Expression plasmids

Plasmid encoding human wild type (wt) glutamate transporter 1 (GLT-1) was purchased from Addgene (#32814, deposited by Susan Amara). The GLT-1 encoding sequence was subcloned into pcDNA^TM^6 V5 Myc (ThermoScientific, Waltham, MA). The STOP codon was left at the end of the GLT-1 encoding sequence leaving the final product untagged.

### Immunoprecipitation (IP)

Cells in culture or mouse brain tissue was lysed in 1% 3-[(3-cholamidopropyl) dimethylammonio]-2-hydroxy-1-propanesulfonate (CHAPSO) (50 mM HEPES, 100 mM NaCl, pH 7.4 and 1% CHAPSO) to keep the PS1 NTF/CTF complex intact, or 1% TX-100 buffer (50 mM HEPES, 100 mM NaCl, pH 7.4 and 1% TX-100) to break the PS1 NTF/CTF complex apart. Both lysis buffers were supplemented with HALT protease and phosphatase inhibitor cocktails (ThermoScientific, Waltham, MA). This was achieved by pipetting the samples up-and-down, passing through a 37-gauge needle and rotating for 1 hour at 4 °C. In order to remove the insoluble fraction, the centrifugation at 14,000xg for 15 minutes was performed. Total protein in the samples was measured using ThermoScientific Pierce BCA Protein Assay (ThermoScientific, Waltham, MA) following the supplier’s protocol.

For the IP procedure, 3 μg of the respective antibody or normal IgG, as a negative control, were added to the aliquots of the supernatant containing equal amount of protein. The samples were then incubated overnight at 4 °C with end-over-end rotation. The antibody-protein complexes were purified by the addition of 30 μl of Protein G Dynabeads (ThermoScientific, Waltham, MA) and a 10-minute incubation of the samples with end-over-end rotation at room temperature. The beads coupled with the complexes were collected using a magnetic tube rack, washed twice with the same lysis buffer that was used to lyse the tissue/cell, and once with the wash buffer containing 50 mM HEPES, 100 mM NaCl, pH7.4. The elution was performed by boiling the samples for 5 minutes in 25 μl 2 × NuPAGE^TM^ LDS (lithium dodecyl sulfate) Sample Buffer supplemented with 1 × NuPAGE^TM^ Sample Reducing Agent buffer containing 50  mM dithiothreitol (ThermoScientific, Waltham, MA).

### Western blotting (WB)

To resolve the proteins, the samples were loaded on 4–12% Bis-Tris NuPage polyacrylamide gels (ThermoScientific, Waltham, MA) and transferred to nitrocellulose membranes (GE Healthcare Lifesciences, Pittsburgh, PA) using BioRad system. The detection was performed by immunoblotting with specific primary and corresponding IRdye680/800- or HRP-conjugated secondary antibodies, and developing the membranes using Odyssey Infrared Imaging System (Li-COR, Lincoln, NE) or Pierce ECL Western Blotting Substrate (Pierce, Rockford, IL) and X-ray films (Amersham Hyperfilm ECL). The quantitative analysis of the respective bands’ optical density was performed using ImageStudio Lite Ver 5.2 software.

### Immunocytochemistry (ICC) and immunohistochemistry (IHC)

Fixation of the brain tissue was achieved by 48-hour incubation of the freshly dissected mouse brains in 15% glycerol, 4% paraformaldehyde (PFA) solution. The brains were sectioned into 35 μm-thick sections on a Leica freezing microtome (Leica SM 2000R, Bannockburn, IL) and used for immunostaining and Förster resonance energy transfer (FRET) analysis. *In vitro* cultured cells were fixed by 15-minute incubation with 4% PFA. Following the fixation, the tissue or cells were permeabilized using 0.4% TX-100 or 0.1% TX-100, respectively. To block non-specific binding of the antibodies, the samples were incubated with 5% normal goat serum (Jackson ImmunoResearch Labs, West Grove, PA) for 1 hour, followed by overnight incubation with respective primary antibodies. Excess antibodies were washed off and the corresponding Alexa Fluor 488- or Cy3-conjugated secondary antibodies were applied for 1 hour at room temperature. The slides were mounted with VectaShield mounting medium (Vector Laboratories, Inc., Burlingame, CA).

### Antibodies

The following primary antibodies were used: anti-GLT-1 raised against the peptide within 30 C-terminal amino acids from the canonical GLT-1 sequence (AB1783, EMD Millipore, Temecula, CA), and anti-GLT-1 raised against the peptide corresponding to residues 550 to the C-terminus of rat GLT-1 (ab41621, Abcam, Cambridge, MA), both recognizing GLT-1a. The GLT-1 antibody from Abcam was used in the co-IP experiments and the antibody from EMD Millipore for the immunofluorescent staining. Anti-GLAST (MABN794, Millipore, Temecula, CA); anti-EAAT3 (MAB1587, Millipore, Temecula, CA); anti-Na^+^/K^+^-ATPase (05-369, Millipore, Temecula, CA); anti-PS1 loop raised against the loop domain between transmembrane domains 6 and 7 of PS1, clone APS18 (ab15458, Abcam, Cambridge, MA) and anti-PS1 CT raised against the C-terminus of PS1 (mAb5643, Cell Signaling Technology, Danvers, MA and P7854, Sigma-Aldrich, St. Louis, MO). Alexa Fluor 488 (ThermoScientific, Waltham, MA) and Cy3-conjugated secondary antibodies (Jackson ImmunoResearch, West Grove, PA) were applied for the microscopy imaging and IRDye680/800- (Li-COR, Lincoln, NE) labeled ones were used for western blotting.

### Spectral Förster resonance energy transfer (FRET)

Spectral FRET assay was conducted as described previously^31,60^. Briefly, the donor Alexa Fluor 488 was exited with the Argon laser at 488 nm. The emitted fluorescence was simultaneously detected in two channels: at 513 ± 10.57 nm and 565 ± 10.57 nm spectral bandwidth of the Metadector on the Zeiss LSM510 confocal microscope. The images were acquired using Zeiss 25x/0.8 Corr DIC objective and ZEN 2009 software. For the analysis, ImageJ 1.46c software was used. Individual cells were outlined as regions of interests (ROIs) and their intensity in the 565 nm and 513 nm channels was measured. The ratio of the fluorescence intensity in the 565 nm (red, R) channel to the intensity in the 513 nm (green, G) channel was used as readout of the FRET efficiency (R/G ratio). The increase in the R/G ratio correlates with the increase in proximity between the fluorophores.

### Fluorescence lifetime imaging microscopy (FLIM)

FLIM assay was conducted as described previously^31,60^. Briefly, cells were immunostained with anti-GLT-1 (Millipore, AB1783) and anti-PS1 antibodies. Corresponding secondary antibodies conjugated with Alexa Fluor 488 (AF488) and Cy3 fluorophores were used as the donor and acceptor, respectively. The sample where the anti-PS1 antibody was omitted was used as a negative control to record the baseline lifetime (*t*1) of the donor fluorophore. A femtosecond-pulsed Chameleon Ti:Sapphire laser (Coherent Inc., Santa Clara, CA) at 850 nm was used for two-photon fluorescence excitation. AF488 fluorescence was acquired using emission filter centered at 515/30 nm. The donor fluorophore lifetimes were measured with a high-speed photomultiplier tube (MCP R3809; Hamamatsu, Bridgewater, NJ) and a fast time-correlated single-photon counting acquisition board (SPC-830; Becker & Hickl, Berlin, Germany). The data were analyzed using SPCImage software (Becker and Hickl, Berlin, Germany). The AF488 lifetimes were calculated by fitting raw data to the single-exponential (AF488 negative control) or multi-exponential (AF488- and Cy3-double immunostained sample) fluorescence decay curves. The *t*_*2*_ lifetime values that are shorter than *t*_1_ indicate presence of the FRET, i.e., less than 5-10 nm distance between the donor and acceptor fluorophores. The calculated lifetimes were displayed on a 128 × 128 pixel matrix to create pseudo-coloured images.

### Statistics

Statistical analysis was performed using Microsoft Office Excel 2007 or GraphPad® Prism 5 (GraphPad Prism Software inc., La Jolla, CA). To determine the Gaussian distribution of the data and the variance equality, the D’Agostino & Pearson omnibus normality test or the F statistics calculations were applied. A standard two-tailed unpaired Student’s t-test was applied to analyze the data, meeting the criteria for Gaussian distribution and equal variance. A p-value of <0.05 was considered a predetermined threshold for statistical significance.

### Data availability

The datasets generated or analyzed during this study are included in this published article and available in the IMEx consortium - IntAct [X] repository [IM-25035].

## Electronic supplementary material

Supplementary information
