# Supplementary material for: Novel interaction between Alzheimer’s disease-related protein presenilin 1 and glutamate transporter 1
Source: Sci Rep. 2018 Jun 7;8:8718. doi: 10.1038/s41598-018-26888-2 (PMC5992168; doi:10.1038/s41598-018-26888-2)

## Supplementary information

### TITLE

Novel interaction between Alzheimer's disease-related protein presenilin 1 and glutamate transporter 1

### AUTHORS

Zoltowska Katarzyna Marta[*], Maesako Masato[*], Meier Joshua, Berezovska Oksana[#]

* Contributed equally

[#] Corresponding author

**ENTIRE WESTERN BLOT GELS**

**Figure 1A**

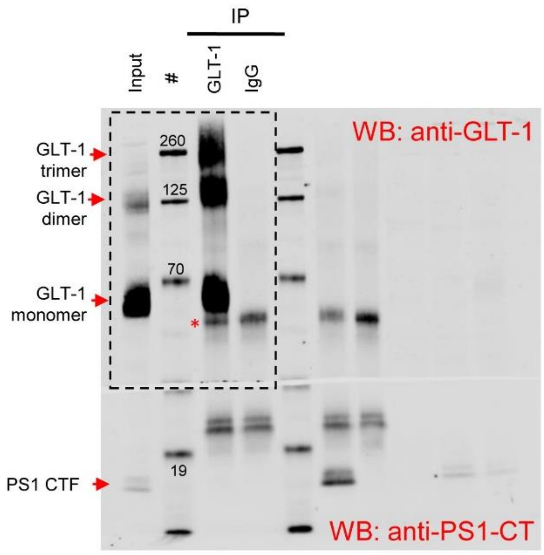


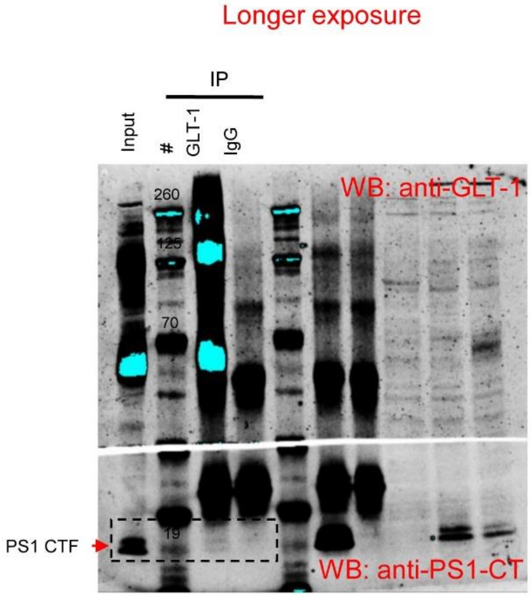


**Figure 1C**

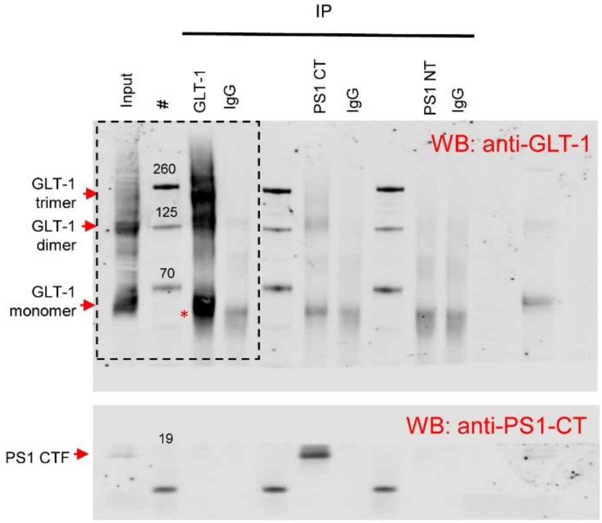


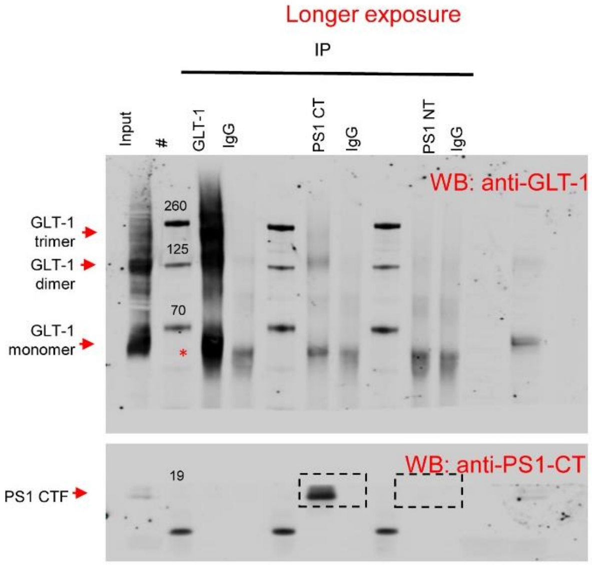

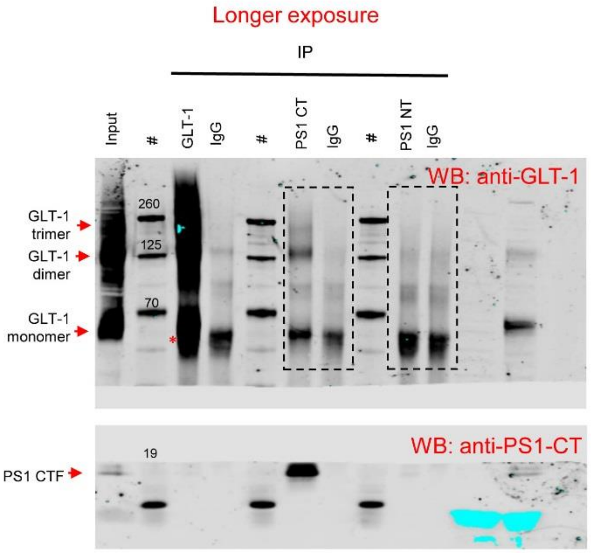
Longer exposure
IP
Input
#
GLT-1
IgG
#
PS1 CT
IgG
#
PS1 NT
IgG
WB: anti-GLT-1
GLT-1 trimer
GLT-1 dimer
GLT-1 monomer
260
125
70
*
WB: anti-PS1-CT
PS1 CTF
19

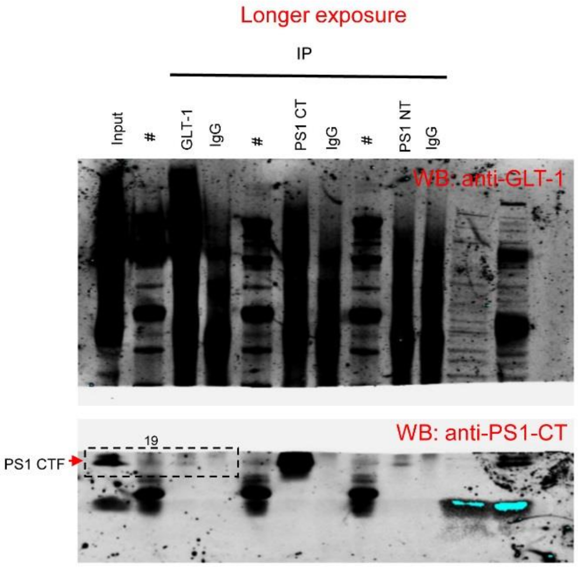
Longer exposure
IP
Input
#
GLT-1
IgG
#
PS1 CT
IgG
#
PS1 NT
IgG
WB: anti-GLT-1
WB: anti-PS1-CT
PS1 CTF
19

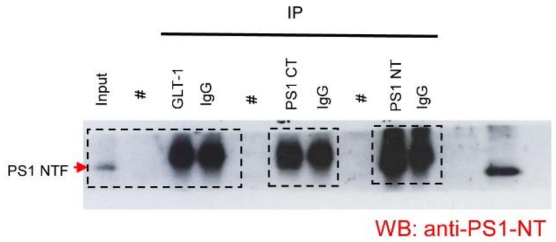
IP
Input
#
GLT-1
IgG
#
PS1 CT
IgG
#
PS1 NT
IgG
PS1 NTF
WB: anti-PS1-NT

**Figure 2A**

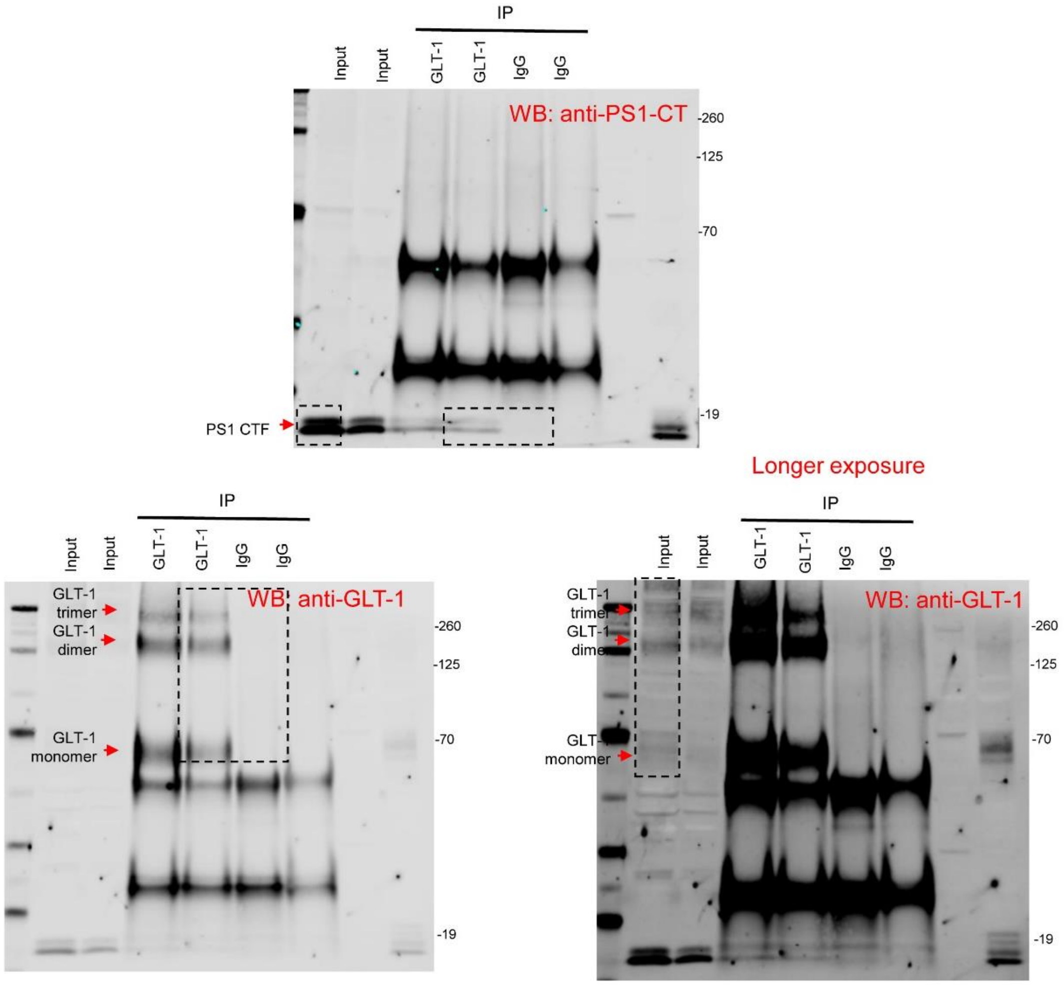

IP
Input
Input
GLT-1
GLT-1
IgG
IgG
WB: anti-PS1-CT
-260
-125
-70
-19
PS1 CTF
Longer exposure
IP
Input
Input
GLT-1
GLT-1
IgG
IgG
GLT-1 trimer
GLT-1 dimer
GLT-1 monomer
WB: anti-GLT-1
-260
-125
-70
-19
IP
Input
Input
GLT-1
GLT-1
IgG
IgG
GLT-1 trimer
WB: anti-GLT-1
-260
-125
-70
-19

**Figure 2D**

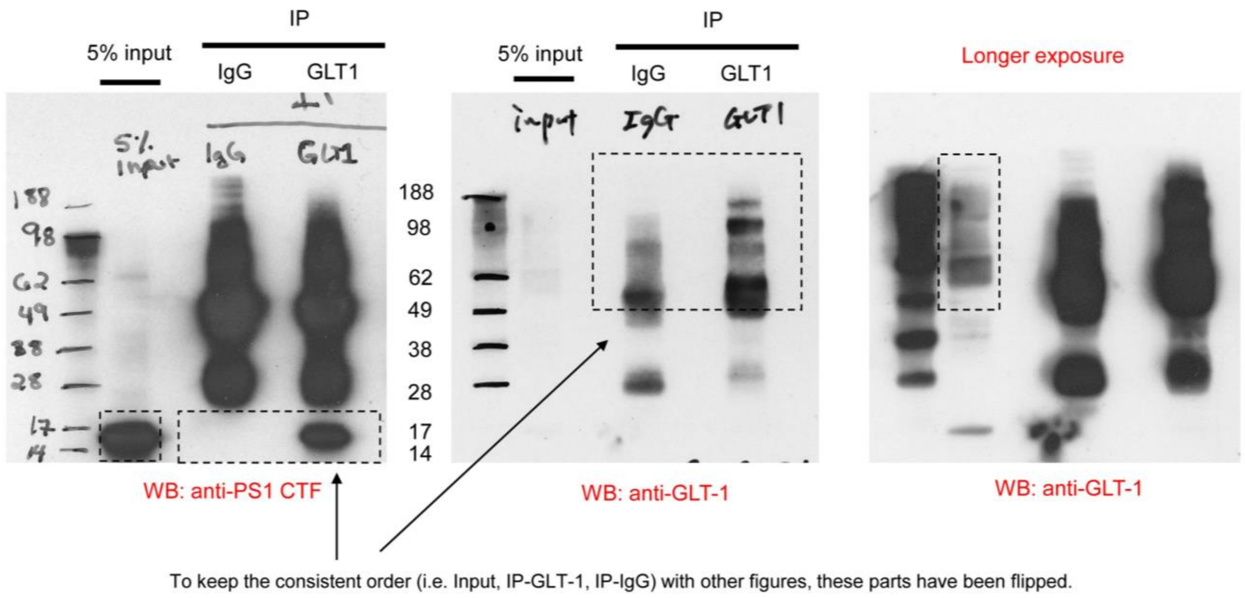


**Figure 3A**

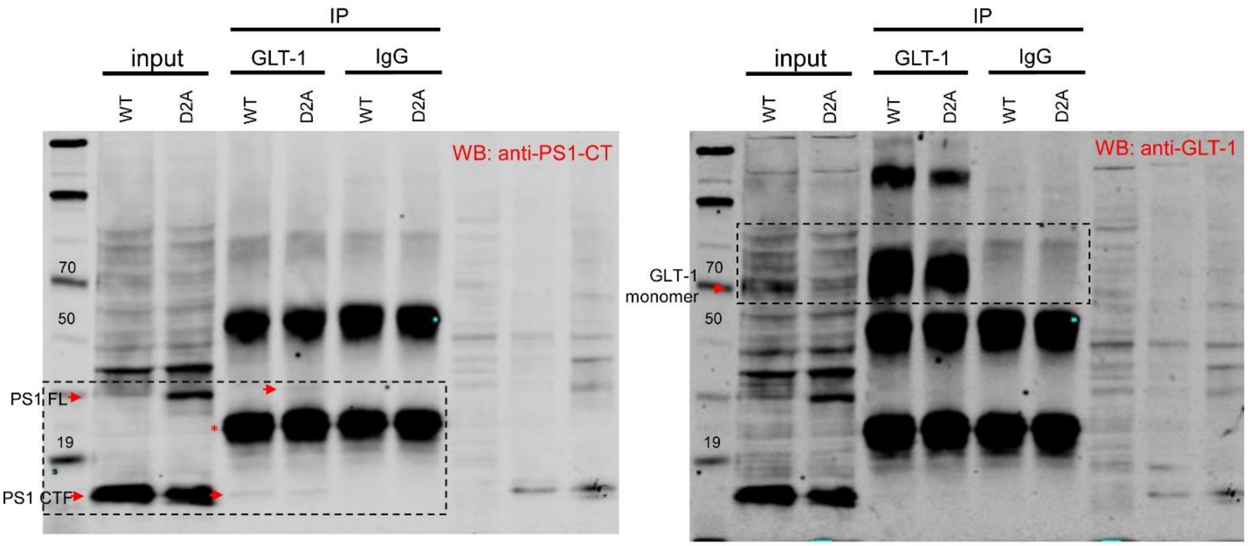

Supplement: Supplementary file 1 — Supplementary information [file 41598_2018_26888_MOESM1_ESM.pdf]
